# Ventricular Septal Defect in Delayed Presentation of ST-Elevation Myocardial Infarction (STEMI) Secondary to COVID-19 Pandemic

**DOI:** 10.7759/cureus.17913

**Published:** 2021-09-12

**Authors:** Pujon Purkayastha, Muhammad J Iftikhar, Maciej Kostrubiec

**Affiliations:** 1 Cardiology, Broomfield Hospital, Chelmsford, GBR

**Keywords:** cardiovascular medicine. hypertensive heart disease. cardio-oncology. cardiac mri, echocardiography in cardio-oncology, cardio, cardio vascular disease, cardio thoracic surgery, public, public health and safety

## Abstract

Like many other countries at the moment, the United Kingdom (UK) is currently under national lockdown due to the coronavirus disease 2019 (COVID-19) pandemic. An unfortunate consequence of such social isolation measures is that patients with genuine acute medical emergencies may not present to a hospital in a timely manner. We present such a scenario, whereby a patient had a delayed presentation of ST-elevation myocardial infarction (STEMI) due to fear of breaching COVID-19 lockdown rules. As a result of the patient presenting well outside the optimal treatment window, her STEMI was complicated by a severe ventricular septal defect (VSD). We discuss how the COVID-19 pandemic has influenced the nature and management of STEMIs and associated issues.

## Introduction

Ischaemic heart disease is one of the leading causes of mortality worldwide and is associated with a range of common diseases, including diabetes and hypertension [1]. An ST-elevation myocardial infarction (STEMI) is the most severe form of acute coronary syndrome whereby there is usually total occlusion of one of the main coronary vessels. Given the acute nature of a STEMI, it is of no surprise that it can be associated with some severe complications. Ventricular septal defects (VSDs) are one such complication, whereby the interventricular septum ruptures leaving a physical connection between the left and right ventricles. VSDs are a rare complication with some recent studies citing a STEMI complication rate of only 0.3% [2]. The risk of developing a significant VSD increases with time making late STEMI presentations a particular risk factor for this often fatal complication. 

## Case presentation

This case study is about an 86-year-old lady with a background of hypertension, atrial fibrillation, and chronic obstructive pulmonary disease, who presented to the emergency department complaining of chest pain which radiated to her left shoulder and jaw. The patient had been experiencing severe symptoms for the preceding three days, but did not immediately come to a hospital to seek medical attention. This delay in seeking treatment is largely attributable to the current coronavirus disease 2019 (COVID-19) pandemic, which is causing an ongoing national lockdown in the United Kingdom (UK). The patient in this case resides in England.

**Figure 1:**
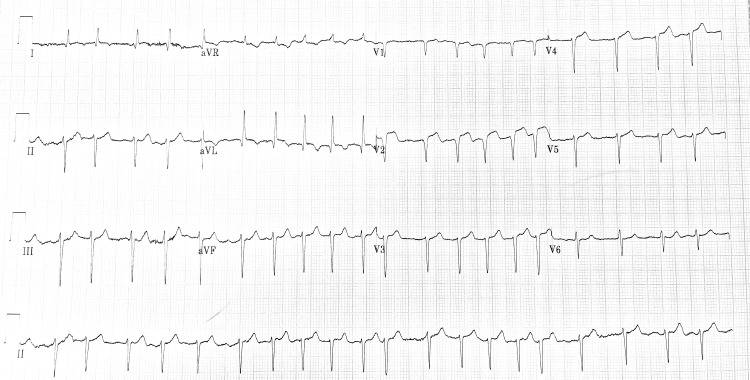
Electrocardiogram (ECG) demonstrating ST-segment elevation predominantly in lead V2

Upon presentation to the emergency department, the patient’s electrocardiogram (ECG) demonstrated atrial fibrillation with anterior ST-segment elevation in keeping with an acute STEMI (Figure 1). The patient’s laboratory blood results were as follows: troponin T 3634 ng/L; D-dimer 728 ng/ml; creatinine 124 umol/L; urea 10.1 mmol/L; potassium 3.8 mmol/L; sodium 134 mmol/L; haemoglobin 128 g/L; platelets 190x109/l; total cholesterol 4.64 mmol/L; high density lipoprotein (HDL) cholesterol 1.58 mmol/L; low density lipoprotein (LDL) cholesterol 3.0 mmol/L; triglycerides 1.22 mmol/L. It is worth noting that the patient, in this case, tested negative for COVID-19 (severe acute respiratory syndrome coronavirus 2 (SARS-CoV-2)) via polymerase chain reaction (PCR) testing. 

**Figure 2:**
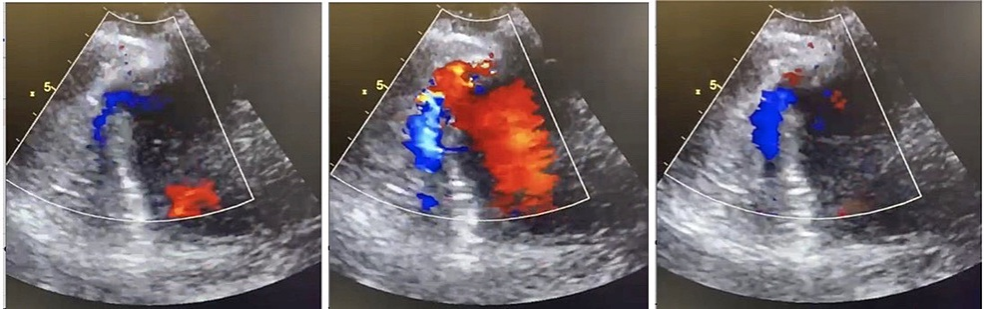
Transthoracic echocardiogram (ECHO) with Doppler colour flow demonstrating ventricular septal defect (VSD)

Despite cautious fluid resuscitation, the patient remained haemodynamically very unstable with a systolic blood pressure hovering between 60-80 mmHg. On examination, the patient had a prominent pansystolic murmur on auscultation (loudest over the left sternal edge), with bibasal crackles on auscultation of her lung fields. We thus proceeded to perform a bedside trans-thoracic echocardiogram (ECHO), which revealed the cause of the cardiovascular compromise to be a large ventricular septal perforation (Figure 2). 

The case was immediately discussed with the regional cardiothoracic centre, and the cardiology team felt that surgical intervention would not be in the patient’s best interests given her advanced age and poor physiological reserve. The patient did receive medical management for her STEMI, which included dual-antiplatelet therapy, a low-molecular-weight heparin injection, and a low dose beta-blocker in combination with an ace-inhibitor. After extensive discussion with the patient and her family, the cardiology team explained that unfortunately, this was a catastrophic complication of a large myocardial infarct and likely represents a terminal event. A joint decision was therefore made to commence the patient on a palliative care pathway.

## Discussion

Impact of COVID-19 pandemic on STEMIs

Towards the end of the year 2019, a novel coronavirus caused by SARS-CoV-2 (termed COVID-19) emerged as a highly contagious disease with the potential to cause acute respiratory failure and ultimately death. Within just a few months after its outbreak in China, the World Health Organisation (WHO) declared COVID-19 a global pandemic. This sequentially triggered numerous countries to establish forms of national lockdown in an attempt to limit spread of the disease. The UK has been through various phases of lockdown, and government advice throughout these periods has urged the public to avoid unnecessary travel and visitation to hospitals. Whilst this was largely an attempt to reduce pressure for struggling frontline healthcare services, an unfortunate consequence has been towards patients with genuine medical emergencies who either do not present to a hospital, or have significant delays in seeking treatment. 

Given that ischaemic heart disease is amongst the most prevalent medical conditions worldwide, it is of little surprise that the COVID-19-induced national lockdowns have caused a dramatic reduction in the rate of hospital admissions related to acute coronary syndrome [3,4]. There are clear guidelines for the timely management of patients presenting with a STEMI, and late presentations are notoriously associated with poor treatment outcomes and higher complication rates. A global survey published by the European Society of Cardiology has highlighted that during this pandemic, greater than 40% of patients with an acute STEMI presented beyond the optimal timeframe for consideration of primary percutaneous coronary intervention (PCI) or even thrombolysis [3]. 

It is also worth noting that numerous studies have now explored the potentially damaging consequences of social distancing and isolation on cardiovascular health and overall mortality [5]. Some studies have even stipulated molecular mechanisms, for instance, activation of the hypothalamic-pituitary-adrenocortical (HPA) axis, which may underlie the increased risk towards cardiovascular disease [6]. Social isolation and loneliness have a well-established association with higher mortality and this effect has been comparable to smoking 15 cigarettes/day [6] and can even exceed the risk of leading a sedentary lifestyle [7]. It is prudent that any national guidelines put in place take into account the negative impact social isolation may have on vulnerable groups of people. Furthermore, measures to combat the sense of loneliness can prove to play a key role in reducing the chances of developing an acute myocardial infarction. 

Additional complications of COVID-19 

Although the patient in this case study tested negative for the presence of COVID-19, there are some unfortunate patients who present with an acute myocardial infarction alongside the coronavirus illness. It is now well recognised that patients inflicted with COVID-19 have a tendency to be in a procoagulant state, whereby they are more likely to suffer from venous and arterial vascular thrombosis [8]. In addition to a higher risk of developing an acute myocardial infarction, these patients are also more likely to develop pulmonary emboli and inflammatory myocarditis, which may clinically present in a similar fashion to a STEMI [9]. This inevitably decreases the diagnostic certainty, and can pose issues when deciphering optimal management of such patients. Furthermore, even if patients confirmed to have a STEMI undergo timely primary PCI, concerns have been raised surrounding the decreased subsequent coronary blood flow as a result of the procoagulant nature of COVID-19 [10]. 

COVID-19 patients with significant cardiovascular comorbidities such as hypertension are more likely to require invasive ventilation in an intensive care setting, which in itself is associated with a poor prognosis [11]. A case series including 5700 patients with COVID-19 carried out in the United States (US) has found that cardiovascular risk factors were amongst the most common comorbidities. The leading association was with hypertension (56.6%), followed by obesity (41.7%) and diabetes (33.8%) [12].

VSDs complicating STEMIs

VSDs remain a rare phenomenon with some recent studies citing a STEMI complication rate of 0.3% [2]. It is worth noting that this complication rate has decreased over the past couple of decades, largely as a consequence of early reperfusion therapy [13]. Nevertheless, VSDs complicating STEMIs continue to carry a high mortality rate ranging consistently above 50% even after surgical repair [13]. Mortality is of particular concern when patients present with cardiogenic shock and cardiac arrest [2], and is significantly higher in patients of advanced age [14]. It is worth noting that the female sex has also been independently associated with a higher mortality rate [15]. Other severe complications associated with STEMIs include ventricular free wall rupture and papillary muscle rupture. Undoubtedly, these should remain high on the list of differential diagnoses in patients with a delayed presentation of a STEMI, particularly when associated with haemodynamic instability. 

## Conclusions

In this case study, we have demonstrated the potentially devastating impact that COVID-19-induced national lockdowns may have on patients experiencing acute coronary syndrome. The fear of breaching lockdown rules coupled with social isolation measures creates the possibility of patients not presenting to a hospital in a timely manner, despite requiring immediate medical attention. We hope further government guidance surrounding any potential national lockdown adopts measures to minimise the risk towards acutely unwell patients.

## References

[1] Gupta R, Wood DA (2019). Primary prevention of ischaemic heart disease: populations, individuals, and health professionals. Lancet.

[2] Singh V, Rodriguez AP, Bhatt P (2017). Ventricular septal defect complicating st-elevation myocardial infarctions: a call for action. Am J Med.

[3] Pessoa-Amorim G, Camm CF, Gajendragadkar P (2020). Admission of patients with STEMI since the outbreak of the COVID-19 pandemic: a survey by the European Society of Cardiology. Eur Heart J Qual Care Clin Outcomes.

[4] Zitelny E, Newman N, Zhao D (2020). STEMI during the COVID-19 pandemic - an evaluation of incidence. Cardiovasc Pathol.

[5] Leigh-Hunt N, Bagguley D, Bash K, Turner V, Turnbull S, Valtorta N, Caan W (2017). An overview of systematic reviews on the public health consequences of social isolation and loneliness. Public Health.

[6] Xia N, Li H (2018). Loneliness, social isolation, and cardiovascular health. Antioxid Redox Signal.

[7] Holt-Lunstad J, Smith TB, Layton JB (2010). Social relationships and mortality risk: a meta-analytic review. PLoS Med.

[8] Becker RC (2020). COVID-19 update: Covid-19-associated coagulopathy. J Thromb Thrombolysis.

[9] Guzik TJ, Mohiddin SA, Dimarco A (2020). COVID-19 and the cardiovascular system: implications for risk assessment, diagnosis, and treatment options. Cardiovasc Res.

[10] Jain V, Gupta K, Bhatia K (2021). Management of STEMI during the COVID-19 pandemic: lessons learned in 2020 to prepare for 2021. Trends Cardiovasc Med.

[11] Driggin E, Madhavan MV, Bikdeli B (2020). Cardiovascular considerations for patients, health care workers, and health systems during the COVID-19 pandemic. J Am Coll Cardiol.

[12] Richardson S, Hirsch JS, Narasimhan M (2020). Presenting characteristics, comorbidities, and outcomes among 5700 patients hospitalized with COVID-19 in the New York City area. JAMA.

[13] Goldsweig AM, Wang Y, Forrest JK (2018). Ventricular septal rupture complicating acute myocardial infarction: incidence, treatment, and outcomes among medicare beneficiaries 1999-2014. Catheter Cardiovasc Interv.

[14] Pradhan A, Jain N, Cassese S (2018). Incidence and predictors of 30-day mortality in patients with ventricular septal rupture complicating acute myocardial infarction. Heart Asia.

[15] Hua K, Peng Z, Yang X (2021). Long-term survival and risk factors for post-infarction ventricular septal rupture. Heart Lung Circ.

